# Early Integrated Palliative Care in Patients With Advanced Cancer: A Randomized Clinical Trial

**DOI:** 10.1001/jamanetworkopen.2024.26304

**Published:** 2024-08-08

**Authors:** EunKyo Kang, Jung Hun Kang, Su-Jin Koh, Yu Jung Kim, Seyoung Seo, Jung Hoon Kim, Jaekyung Cheon, Eun Joo Kang, Eun-Kee Song, Eun Mi Nam, Ho-Suk Oh, Hye Jin Choi, Jung Hye Kwon, Woo Kyun Bae, Jeong Eun Lee, Kyung Hae Jung, Young Ho Yun

**Affiliations:** 1 National Cancer Control Institute, National Cancer Center, Goyang, Republic of Korea; 2 Department of Family Medicine, National Cancer Center, Goyang, Republic of Korea; 3 Department of Internal Medicine, Gyeongsang National University, Jinju, Republic of Korea; 4 Department of Hematology and Oncology, Ulsan University Hospital, Ulsan University College of Medicine, Ulsan, Republic of Korea; 5 Department of Internal Medicine, Seoul National University Bundang Hospital, Seoul National University College of Medicine, Seongnam, Republic of Korea; 6 Department of Oncology, Asan Medical Center, Ulsan University College of Medicine, Seoul, Republic of Korea; 7 Department of Hemato-Oncology, CHA Bundang Medical Center, CHA University, Seongnam, Republic of Korea; 8 Department of Hemato-Oncology, Korea University Guro Hospital, Korea University College of Medicine, Seoul, Republic of Korea; 9 Department of Internal Medicine, Jeonbuk National University Medical School, Jeonju, Republic of Korea; 10 Department of Internal Medicine, Ewha Womans University College of Medicine, Seoul, Republic of Korea; 11 Division of Hemato-Oncology, Department of Internal Medicine, GangNeung Asan Hospital, University of Ulsan College of Medicine, Gangneung, Republic of Korea; 12 Division of Medical Oncology, Department of Internal Medicine, Yonsei Cancer Center, Yonsei University College of Medicine, Seoul, Republic of Korea; 13 Department of Internal Medicine, College of Medicine, Chungnam National University College of Medicine, Daejeon, South Korea; 14 Department of Internal Medicine, Chungnam National University Sejong Hospital, Sejong, Republic of Korea; 15 Daejeon Regional Cancer Center, Daejeon, Republic of Korea; 16 Division of Hematology-Oncology, Department of Internal Medicine, Chonnam National University Medical School and Hwasun Hospital, Hwasun, Republic of Korea; 17 Department of Family Medicine, Seoul National University Hospital, Seoul, Republic of Korea; 18 Department of Human System Medicine, Seoul National University College of Medicine, Seoul, Republic of Korea

## Abstract

**Importance:**

Limited data suggest that early palliative care (EPC) improves quality of life (QOL) and survival in patients with advanced cancer.

**Objective:**

To evaluate whether comprehensive EPC improves QOL; relieves mental, social, and existential burdens; increases survival rates; and helps patients develop coping skills.

**Design, Setting, and Participants:**

This nonblinded randomized clinical trial (RCT) recruited patients from 12 hospitals in South Korea from September 2017 to October 2018. Patients aged 20 years or older with advanced cancer who were not terminally ill but for whom standard chemotherapy has not been effective were eligible. Participants were randomized 1:1 to the control (receiving usual supportive oncological care) or intervention (receiving EPC with usual oncological care) group. Intention-to-treat data analysis was conducted between September and December 2022.

**Interventions:**

The intervention group received EPC through a structured program of self-study education materials, telephone coaching, and regular assessments by an integrated palliative care team.

**Main Outcomes and Measures:**

The primary outcome was the change in overall QOL score (assessed with the European Organization for Research and Treatment of Cancer Quality of Life Questionnaire Core 15 Palliative Care) from baseline to 24 weeks after enrollment, with evaluations also conducted at 12 and 18 weeks. Secondary outcomes were social and existential burdens (assessed with the McGill Quality of Life Questionnaire) as well as crisis-overcoming capacity and 2-year survival.

**Results:**

A total of 144 patients (83 males [57.6%]; mean [SD] age, 60.7 (7.2) years) were enrolled, of whom 73 were randomized to the intervention group and 71 to the control group. The intervention group demonstrated significantly greater changes in scores in overall health status or QOL from baseline, especially at 18 weeks (11.00 [95% CI, 0.78-21.22] points; *P* = .04; effect size = 0.42). However, at 12 and 24 weeks, there were no significant differences observed. Compared with the control group, the intervention group also showed significant improvement in self-management or coping skills over 24 weeks (20.51 [95% CI, 12.41-28.61] points; *P* < .001; effect size = 0.93). While the overall survival rate was higher in the intervention vs control group, the difference was not significant. In the intervention group, however, those who received 10 or more EPC interventions (eg, telephone coaching sessions and care team meetings) showed a significantly increased probability of 2-year survival (53.6%; *P* < .001).

**Conclusions and Relevance:**

This RCT demonstrated that EPC enhanced QOL at 18 weeks; however, no significant improvements were observed at 12 and 24 weeks. An increased number of interventions sessions was associated with increased 2-year survival rates in the intervention group.

**Trial Registration:**

ClinicalTrials.gov Identifier: NCT03181854

## Key Points

**Question:**

Does systematic, integrated, early palliative care (EPC) improve quality of life, enhance self-management competency or coping skill, and increase survival rates in patients with advanced cancer but who are not terminally ill for whom standard chemotherapy has not been effective?

**Findings:**

In this randomized clinical trial of 144 patients with advanced cancer, those randomized to the EPC intervention group experienced better quality of life at 18 months, but not at 12 or 24 months, compared with the control group. Patients in the intervention group reported lower existential burden and enhanced coping skills compared with the control group.

**Meaning:**

Findings from this study suggest the need to develop guidelines for systematic EPC to improve its quality and to develop various methods of providing EPC to increase patients’ adherence with interventions.

## Teaser

This randomized clinical trial investigates the change in quality of life, survival rates, and self-management skills associated with an early palliative care program for patients with advanced cancer.

## Introduction

Despite advances in the development of innovative cancer treatment methods, patients with advanced cancer struggle with enormous physical, mental, social, and existential burdens.^1,2,3^ To assess and manage the substantial unmet needs of patients with advanced cancer, the World Health Organization and the American Society of Clinical Oncology recommend early initiation of palliative care (PC), which was traditionally provided toward the end of life only. It is explicitly recommended that PC be integrated early in the disease trajectory for patients with advanced cancer. Early PC (EPC) involves establishing a PC hospital support team, which includes PC specialists.^4^ Accumulating data have demonstrated promising outcomes for quality of life (QOL) and symptom management with the early introduction of PC.^5,6,7,8^ Patients receiving EPC had less aggressive care at the end of life^9^ and showed improvements in overall QOL.^5,10^ In Korea, most patients (84.5%) and caregivers (89.5%) had favorable attitudes toward EPC.^11^

In preceding studies, EPC was defined as being implemented within 8 weeks of an advanced cancer diagnosis.^12^ This EPC approach includes discussing available cancer treatments, building rapport, improving coping abilities and understanding of the disease, and planning for the end of life.^12,13^ In other words, EPC usually focuses on facilitating patient choice by describing practical and achievable treatment goals, providing appropriate information, and assessing the patient’s values and preferences in relation to advanced care planning.^14^ For EPC to work successfully, it is also important to induce patients to develop an active attitude or coping skills so that they can make their own choices through EPC.^15^ However, there are not enough studies on how EPC affects the development of patients’ active attitudes or coping skills. Additionally, there is insufficient research on whether EPC relieves mental, social, and existential burdens. Consequently, current results should be interpreted with caution because of differences in participant populations, interventions, and methods between studies.^16^

In previous studies, EPC did not improve QOL in patients with advanced cancer, or the effect size was small,^17^ as a systematic review found.^18^ The effects of EPC interventions on mortality also were uncertain. Despite the proven benefits of and recommendations for EPC, many patients do not receive PC services or are offered PC late in the disease trajectory.^19^ A patient’s belief that PC is appropriate only after the discontinuation of life-sustaining therapy or a clinician’s misunderstanding due to uncertain evidence about QOL or the development of coping skills serve as a barrier to EPC.^20^

However, PC is a necessary component of comprehensive care for patients with advanced cancer, and it must be incorporated early into the treatment plan. We conducted a randomized clinical trial (RCT) comparing the effects of the early and systematic integration of PC into oncological treatment vs usual oncological care alone on survival, QOL, and coping skills in patients with advanced cancer who were not terminally ill but for whom standard chemotherapy has not been effective. In this study, we assessed the effectiveness of EPC, which included structured consultations by the PC team. Our objective was to evaluate whether comprehensive EPC improves QOL; relieves mental, social, and existential burdens; increases survival rates; and helps patients to develop coping skills.

## Methods

### Study Design and Participants

This nonblinded RCT compared standard supportive oncological care vs early and systematic integrated PC in patients with advanced cancer. Patients who were already receiving or had received PC were not included in this study. The Seoul National University College of Medicine and Hospital Institutional Review Board (IRB) and the IRBs of the 12 participating hospitals approved the study protocol. This research protocol is provided in Supplement 1. Patients provided written informed consent. We followed the Consolidated Standards of Reporting Trials (CONSORT) reporting guideline.

From September 2017 to October 2018, we recruited patients with advanced cancer at 12 hospitals in South Korea. Patients were identified for enrollment by their oncologists, and both outpatients and inpatients were considered for inclusion. Eligible patients were enrolled if they met the following criteria: (1) aged 20 years or older; (2) advanced cancer diagnosis (confirmed histologically or cytologically) that originated from a solid tumor; (3) Eastern Cooperative Oncology Group (ECOG) Performance Status score of 0 to 2 (0 indicating fully active at predisease performance; 1, ambulatory but restricted in physically strenuous activity; 2, not fully ambulatory but lying or sitting less than 50% of the day); (4) estimated life expectancy of 12 months or less as determined by the treating oncologist; and (5) ability to consent to participate in the study. Patients were not eligible if they were unable to speak or read Korean, were in poor health (eg, had symptoms of dyspnea, severe depression, or other psychological problems), were not currently receiving cancer treatment, or had previously received PC.

### Randomization

**Figure 1.**
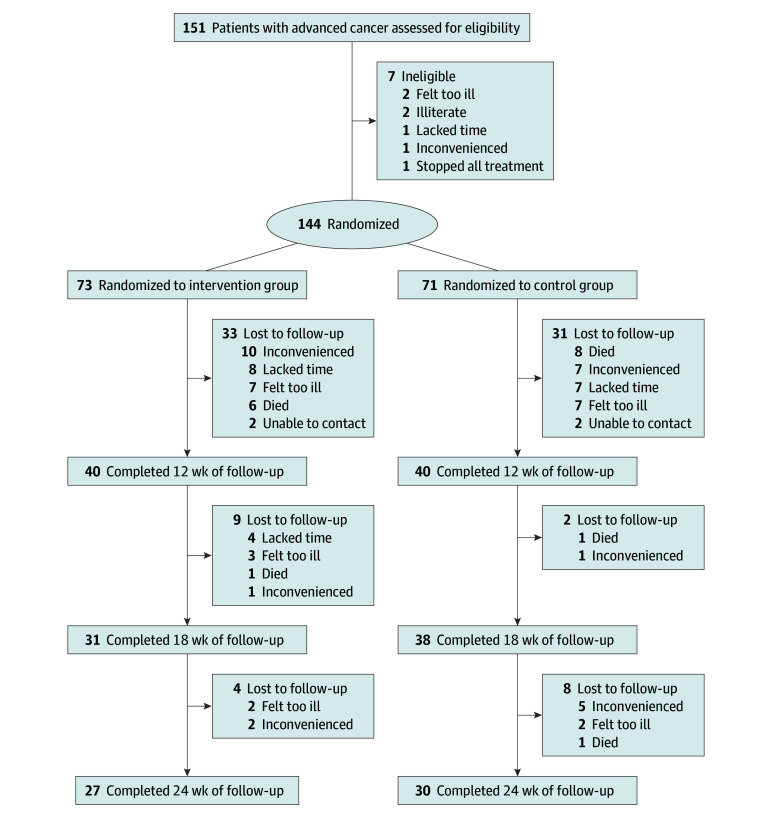
CONSORT Diagram of Patient Flow Through the Trial

Eligible patients were randomly assigned to the control (usual supportive oncological care) or intervention (EPC with usual oncological care) group (Figure 1) in a 1:1 ratio using a computerized random number generator (SAS 9.1.3, PROC PLAN; SAS Institute Inc). To minimize the effects of potential confounding variables, we used a block design to randomize participants into strata defined by cancer type (based on the prevalence of certain cancers in South Korea: breast cancer, gastrointestinal cancer, hepatobiliary cancer, lung cancer, and all other cancers), age (<65 or ≥65 years), and enrollment hospital. The clinical research coordinator enrolled patients as well as obtained patient study numbers and the corresponding treatment allocation from an administrative assistant at the hospital.

### Intervention

Within 3 weeks of randomization to the intervention group, the first meeting with the integrated PC team was held. At the time of the baseline questionnaire completion, patients were provided with self-study educational materials and videos on EPC and advanced care planning, which were developed based on the Smart Management Strategy for Health. During the initial meeting with the PC team, the patient consulted a PC specialist to evaluate their pain and requirements and to develop a PC plan. This plan was then communicated to the entire team, including the health coaches. After the initial meeting with the PC team, telephone coaching was conducted by PC specialists once a week for the first 12 weeks and then biweekly until the end of the study. Outpatient PC consultations were conducted according to the PC plan tailored to the patient’s needs. Once every 3 weeks for 6 months, symptom control and other mental, social, and spiritual problems were evaluated by the PC team for advanced care planning. Symptoms were assessed by PC nurses every 3 weeks using the 13-item symptom scale of the MD Anderson Symptom Inventory^21^ and Patient Health Questionnaire-2.^22^ Patients with high scores were managed by the PC physician.

Patients randomized to the control group received usual oncological care. Palliative care consultations were available to all patients for whom curative treatment was no longer available, and patients were referred to the PC physician for refractory symptoms or end-of-life care. All control group participants received a 23-minute video on cancer pain management and a 26-page companion booklet titled *Controlling Cancer Pain*, which was developed by the National Cancer Center–Korea. All video materials were provided as CDs, and quick response codes were generated for home use.

### Palliative Care Team

The integrated PC team consisted of specialist PC clinicians and professionally trained health coach nurses. Depending on the hospital, some PC teams also included social workers. A specialist PC clinician is a physician who provides PC and has received specialized training in this field. The health coaches are nurses with over 3 years of clinical experience. Their training included 23 hours of offline lectures and 14 hours of coaching practice through teleclasses (ie, conducted over the telephone). Outpatient consultations with PC specialists were based on the PC plan formulated during the initial meeting, tailored to meet the patient’s specific needs.

Health coaches uploaded their telephone consultation records to the website after coaching for patient management by the palliative medical team, and the central institution continually reviewed and provided feedback on the journal content. The telephone consultation record contained a comprehensive assessment of the progress of the patient’s primary symptoms, consultations on the patient’s health issues, workbook details, coach compliments, patient’s thoughts, and overall status of the patient.

### Measures

Demographic information included age, sex, educational level, employment status, household monthly income, and area of residence. Other baseline data included tumor type and stage as well as ECOG Performance Status score.

The primary outcome was the change in overall QOL score from baseline to 24 weeks, evaluated through the global health status or QOL scale of the European Organization for Research and Treatment of Cancer Quality of Life Questionnaire Core 15 Palliative Care (EORTC QLQ-C15-PAL; score range: 0-100, with higher numbers indicating better quality of life). As secondary outcomes, we used the EORTC QLQ-C15-PAL functional and symptoms scales as well as the McGill Quality of Life Questionnaire (MQOL; score range: 0-10, with higher numbers representing better quality of life) functional scales to identify social and existential burdens and crisis-overcoming capacity at baseline and at 12, 18, and 24 weeks. Crisis-overcoming capacity also was evaluated by the Smart Management Strategy for Health Assessment Tool–Short Form (SAT-SF; score range: 0-100, with higher numbers representing better self-management abilities),^23^ an abbreviated 30-item version of the full 91-item SAT. The SAT-SF evaluates core strategies, preparation strategies, and action strategies, with 10 items in each of these 3 areas.

When patients died during follow-up, family caregivers were asked about the time of death and whether the patient used a PC facility (home PC is not available in South Korea) or an intensive care unit to perform a survival analysis. Mortality data were gathered from death records in Statistics Korea (KOSTAT).

### Statistical Analysis

Considering that the intervention group was receiving EPC, we selected 2-year survival as the main survival outcome. Kaplan-Meier curves were used to compare the survival rates between the intervention and control groups 2 years after enrollment. Participants were divided into the fewer than 10 EPC interventions (telephone coaching sessions and care team meetings and assessments) group vs 10 or more interventions group to determine if the survival rate was higher in the group that received more interventions. Patients who were alive at the time of the final follow-up (2 years from the enrollment date) were censored on that date. Based on the estimated regression model, the mean observed changes in score from baseline and adjusted differences between changes in scores at 12, 18, and 24 weeks were analyzed for patients who survived and responded. Effect sizes were calculated as standardized mean differences (Cohen *d*), with effect sizes of at least 0.3 considered clinically relevant.^24^

Two-sided *P* < .05 indicated statistical significance. Intention-to-treat analysis was conducted between September and December 2022, using Stata version 14.1 (StataCorp LLC).

## Results

### Baseline Characteristics and Intervention Participation

A total of 150 patients in South Korea were evaluated for eligibility, of whom 144 were enrolled and 6 were excluded. Among the 144 participants, 73 were randomized to the intervention group and 71 to the control group (Figure 1). Participants consisted of 83 males (57.6%) and 61 females (42.4%), with a mean (SD) age of 60.7 (7.2) years. The majority of participants had an educational level below a high school diploma (68 [47.2%]). In the analysis of baseline sociodemographic characteristics, there were no significant differences between the intervention and control groups in sex, age, educational level, employment status, marital status, household monthly income, or area of residence. There were more males in the control group than in the intervention group, and the mean (SD) age was similar in the 2 groups (60.8 [8.0] years vs 60.6 [9.7] years) (Table 1).

**Table 1.** Sociodemographic Characteristics of the Participants^a^

Characteristic	Participants, No. (%)	
	Intervention group (n = 73)	Control group (n = 71)
Sex		
Male	39 (53.4)	44 (62.0)
Female	34 (46.6)	27 (38.0)
Age, mean (SD), y	60.6 (9.7)	60.8 (8.0)
Educational level		
<High school	31 (42.5)	37 (52.1)
High school diploma	32 (43.8)	23 (32.4)
≥College degree	10 (13.7)	10 (15.5)
Employment status		
Retired	14 (19.2)	7 (9.9)
Employed	6 (8.2)	7 (9.9)
Unemployed	52 (71.2)	56 (78.9)
Student	1 (1.4)	1 (1.4)
Marital status		
Married or with common law partner	53 (72.6)	56 (78.9)
Other^b^	20 (27.4)	15 (21.1)
Household monthly income, US$		
<2000	41 (57.0)	38 (53.5)
2000-3999	22 (30.5)	27 (38.0)
≥4000	9 (12.5)	6 (8.5)
Area of residence		
Rural or suburban	27 (37.0)	31 (43.7)
Urban	46 (63.0)	40 (56.3)
Tumor site		
Breast	10 (13.7)	7 (9.9)
Gastrointestinal	12 (16.4)	14 (19.7)
Hepatobiliary	39 (53.4)	34 (47.9)
Lung	8 (11.0)	10 (14.1)
Other^c^	4 (5.5)	6 (8.5)
ECOG Performance Status score^d^		
0	3 (4.1)	4 (5.6)
1	56 (76.7)	61 (85.9)
2	14 (19.2)	6 (8.5)
EORTC QLQ-C15-PAL, mean (95% CI)		
Global health status or quality of life	57.8 (52.8-62.7)	50.9 (46.2-55.6)
Physical functioning	62.4 (56.7-68.0)	61.8 (56.6-67.0)
Emotional functioning	82.1 (77.0-87.1)	74.6 (69.3-80.0)
Dyspnea	16.4 (10.8-22.1)	20.7 (14.6-26.7)
Pain	22.6 (16.1-29.1)	27.7 (21.1-34.3)
Insomnia	25.1 (18.8-31.4)	30.5 (23.9-37.2)
Appetite loss	29.7 (23.3-36.1)	31.9 (24.8-39.0)
Constipation	26.9 (20.0-33.9)	27.2 (20.3-34.2)
Fatigue	35.2 (30.3-40.0)	38.5 (33.2-43.8)
Nausea and vomiting	11.2 (7.4-15.0)	15.7 (10.2-21.2)
MQOL, mean (95% CI)		
Emotional well-being	6.2 (5.7-6.6)	5.8 (5.3-6.2)
Social support	7.0 (6.5-7.5)	6.4 (6.0-6.9)
Self-management strategies, mean (95% CI)		
SAT-SF total	45.6 (39.7-51.5)	43.7 (38.2-49.2)
SAT-SF core strategy	55.6 (48.7-62.5)	52.7 (46.4-58.9)
SAT-SF preparation strategy	40.9 (34.5-47.4)	41.2 (35.2-47.2)
SAT-SF implementation strategy	40.2 (34.5-45.8)	37.3 (32.0-42.5)

Abbreviations: ECOG, Eastern Cooperative Oncology Group; EORTC QLQ-C15-PAL, European Organization for Research and Treatment of Cancer Quality of Life Questionnaire Core 15 Palliative Care (score range: 0-100, with higher numbers indicating better quality of life); MQOL, McGill Quality of Life Questionnaire (score range: 0-10, with higher numbers representing better quality of life); SAT-SF, Smart Management Strategy for Health Assessment Tool–Short Form (score range: 0-100, with higher numbers representing better self-management abilities).

^a^ In some factors, there may be a missing value due to nonresponse of participants.

^b^ Other marital status includes single, divorced, widowed, and separated.

^c^ Other tumor site includes pancreatic and cervical.

^d^ An ECOG Performance Status score of 0 indicates fully active at predisease performance; 1, ambulatory but restricted in physically strenuous activity; and 2, not fully ambulatory but lying or sitting less than 50% of the day.

Sixty-four patients (44.4%) were lost to follow-up before 3 months, and 80 patients (55.6%) underwent the week-12 evaluation. From that point until 18 weeks after enrollment, another 11 patients were lost to follow-up, and an additional 12 patients were lost to follow-up before week 24, leaving 57 patients (39.6%) who completed the 24-week follow-up (Figure 1).

### Quality of Life

Changes in score from baseline were calculated and compared between the intervention and control groups (Table 2). In the case of global health status or QOL, there were no significant differences between 12 and 24 weeks, but at 18 weeks the difference from the baseline score was significantly higher in the intervention group than in the control group (11.00 [95% CI, 0.78-21.22] points; *P* = .04; effect size = 0.42). In the intervention group, there were significant differences in score changes at 18 weeks for appetite loss (−14.51 [95% CI, −27.57 to −1.45] points; *P* = .03; effect size = −0.42) and constipation (−11.53 [95% CI, −23.37 to −0.30] points; *P* = .04; effect size = −0.41). Significant differences in score changes also were shown at 24 weeks for physical functioning (12.12 [95% CI, 3.31-20.93] points; *P* = .007; effect size = 0.54) and fatigue (−7.84 [95% CI, −13.52 to −1.08] points; *P* = .02; effect size = −0.36). Other QOL indicators did not show significant differences from baseline.

**Table 2.** Mean Observed Score Changes From Baseline and Adjusted Differences in Score Changes vs Baseline in Quality-of-Life Measures by Groups

EORTC QLQ-C15-PAL measure; Time from baseline, wk	Intervention group		Control group		Available case analysis^a^		
	No.	Mean observed score change from baseline (95% CI)	No.	Mean observed score change from baseline (95% CI)	Adjusted difference between score changes (95% CI)	*P* value	Effect size
Global health status or quality of life							
12	40	3.75 (−3.84 to 11.34)	40	1.25 (−5.62 to 8.12)	2.24 (−7.40 to 11.88)	.65	0.10
18	31	11.29 (1.25 to 21.32)	38	2.63 (−5.38 to 10.64)	11.00 (0.78 to 21.22)^b^	.04	0.42
24	27	6.79 (−4.67 to 18.25)	30	−1.67 (−9.73 to 6.40)	6.47 (−4.46 to 17.39)	.25	0.25
Physical functioning							
12	40	3.83 (−1.54 to 9.21)	40	0.67 (−3.98 to 5.32)	2.68 (−5.11 to 10.47)	.50	0.17
18	31	−1.94 (−10.53 to 6.66)	38	−3.86 (−9.57 to 1.85)	2.07 (−6.21 to 10.35)	.63	0.10
24	27	4.20 (−3.72 to 12.11)	30	−7.74 (−16.14 to 0.66)	12.12 (3.31 to 20.93)^b^	.007	0.54
Emotional functioning							
12	40	5.83 (0.07 to 11.6)	40	2.71 (−3.22 to 8.63)	2.89 (−5.21 to 10.99)	.48	0.16
18	31	1.07 (−6.44 to 8.59)	38	−3.73 (−9.41 to 1.96)	2.35 (−6.25 to 10.96)	.59	0.12
24	27	5.56 (0.38 to 10.73)	30	−2.50 (−13.47 to 8.47)	6.03 (−3.18 to 15.24)	.20	0.26
Dyspnea							
12	40	2.50 (−4.90 to 9.91)	40	0.00 (−7.23 to 7.24)	3.50 (−6.47 to 13.46)	.49	0.15
18	31	1.08 (−6.32 to 8.47)	38	3.51 (−4.46 to 11.48)	−3.85 (−14.44 to 6.73)	.48	−0.17
24	27	2.47 (−7.16 to 12.09)	30	1.08 (−10.94 to 13.09)	2.66 (−8.61 to 13.92)	.64	0.09
Pain							
12	40	−0.83 (−8.74 to 7.08)	40	−0.83 (−7.55 to 5.88)	1.69 (−8.02 to 11.40)	.73	0.07
18	31	0.00 (−9.73 to 9.73)	38	7.89 (−0.23 to 16.02)	−5.30 (−15.62 to 5.02)	.31	−0.21
24	27	−3.70 (−10.86 to 3.46)	30	2.22 (−8.21 to 12.65)	−2.98 (−14.03 to 8.07)	.60	−0.13
Insomnia							
12	40	−4.17 (−12.59 to 4.26)	40	−5.00 (−12.84 to 2.84)	−1.09 (−12.75 to 10.57)	.86	−0.04
18	31	−1.08 (−11.77 to 9.62)	38	4.39 (−5.86 to 14.63)	−6.88 (−19.26 to 5.50)	.28	−0.23
24	27	3.70 (−8.61 to 16.02)	30	−5.38 (−17.23 to 6.48)	3.43 (−9.75 to 16.61)	.61	0.11
Appetite loss							
12	40	−8.33 (−16.95 to 0.29)	40	3.33 (−6.26 to 12.93)	−6.93 (−19.24 to 5.38)	.27	−0.24
18	31	−8.60 (−19.97 to 2.77)	38	11.40 (−0.35 to 23.15)	−14.51 (−27.57 to −1.45)^b^	.03	−0.42
24	27	−7.41 (−18.58 to 3.77)	30	0.01 (−11.81 to 11.81)	−2.48 (−16.37 to 11.42)	.73	−0.08
Constipation							
12	40	−5.83 (−16.04 to 4.38)	40	0.83 (−4.82 to 6.49)	−2.74 (−13.88 to 8.40)	.63	−0.11
18	31	−4.30 (−14.65 to 6.05)	38	9.65 (0.86 to 18.44)	−11.53 (−23.37 to −0.30)^b^	.04	−0.41
24	27	−6.17 (−18.86 to 6.52)	30	4.44 (−4.04 to 12.93)	−5.60 (−18.28 to 7.07)	.39	−0.20
Fatigue							
12	40	−6.67 (−13.15 to −0.19)	40	1.67 (−4.49 to 7.83)	−6.37 (−15.14 to 2.40)^b^	.15	−0.32
18	31	−3.23 (−10.76 to 4.30)	38	4.09 (−3.08 to 11.27)	−6.21 (−15.52 to 3.10)	.19	−0.29
24	27	−7.01 (−14.82 to 0.82)	30	0.74 (−8.04 to 9.52)	−7.84 (−13.52 to −1.08)^b^	.02	−0.36
Nausea and vomiting							
12	40	−2.92 (−8.82 to 2.98)	40	−0.83 (−6.98 to 5.32)	−0.95 (−8.52 to 6.63)	.81	−0.05
18	31	−2.69 (−7.18 to 1.80)	38	4.39 (−2.55 to 11.33)	−4.53 (−12.57 to 3.51)	.27	−0.25
24	27	−4.32 (−13.93 to 5.28)	30	−1.61 (−5.89 to 2.67)	−1.39 (−9.95 to 7.17)	.75	−0.08

Abbreviation: EORTC QLQ-C15-PAL, European Organization for Research and Treatment of Cancer Quality of Life Questionnaire Core 15 Palliative Care.

^a^ Differences in score changes between groups and associated tests of effect were estimated by regression, adjusting for baseline covariates.

^b^ Effect sizes of at least 0.3 as standardized mean differences (Cohen *d*) were considered clinically relevant.

### Social and Existential Burdens and Self-Management Strategies

In this study, MQOL’s existential well-being and social support indices were evaluated (eTable in Supplement 2). In an analysis of the change in scores from baseline in the existential well-being index, the difference between the intervention and control groups was significant at the 24-week follow-up (0.82 [95% CI, 0.03-1.67] points; *P* = .05; effect size = 0.40). There was no significant difference in social support.

Self-management competency or coping skill was divided into core strategy, preparation strategy, and implementation strategy for the evaluation (Table 3). The total self-management strategies score, which is the sum of the strategies, showed a significant difference in change in scores from baseline. The range of change in scores up to 24 weeks was significantly greater in the intervention group than in the control group (20.51 [95% CI, 12.41-28.61] points; *P* < .001; effect size = 0.93). All 3 strategies showed significant differences in the range of change in scores up to 24 weeks (core: 17.16 [95% CI, 6.14-28.18] points, *P* = .002, effect size = 0.63; preparation: 23.44 [95% CI, 14.40-32.48] points, *P* < .001, effect size = 1.01; implementation: 21.27 [95% CI, 12.37-30.17] points; *P* < .001, effect size = 0.87).

**Table 3.** Score Changes vs Baseline and Adjusted Differences Between Score Changes in Self-Management Strategies by Groups

SAT-SF measure; time from baseline, wk	Intervention group		Control group		Available case analysis^a^		
	No.	Mean observed score change from baseline (95% CI)	No.	Mean observed score change from baseline (95% CI)	Adjusted difference between score changes (95% CI)	*P* value	Effect size
Total							
12	40	5.63 (−0.48 to 11.73)	40	−3.34 (−7.67 to 0.99)	9.60 (2.41 to 16.79)^b^	.009	0.57
18	31	7.74 (−1.68 to 17.17)	38	−4.50 (−8.86 to −0.15)	10.98 (3.42 to 18.55)^b^	.004	0.53
24	27	18.36 (10.28 to 26.44)	30	−3.16 (−10.12 to 3.81)	20.51 (12.41 to 28.61)^b^	<.001	0.93
Core strategy							
12	40	3.80 (−5.32 to 12.92)	40	−4.74 (−11.41 to 1.93)	9.11 (−0.68 to 18.90)^b^	.07	0.37
18	31	0.09 (−12.85 to 13.03)	38	−6.41 (−11.50 to −1.33)	5.20 (−5.09 to 15.50)	.32	0.20
24	27	13.09 (1.89 to 24.30)	30	−5.44 (−14.34 to 3.46)	17.16 (6.14 to 28.18)^b^	.002	0.63
Preparation strategy							
12	40	6.02 (−0.10 to 12.15)	40	−5.36 (−10.56 to −0.16)	12.16 (4.13 to 20.19)^b^	.003	0.67
18	31	10.47 (0.61 to 20.32)	38	−6.11 (−11.74 to −0.49)	15.40 (6.96 to 23.85)^b^	<.001	0.66
24	27	18.68 (10.39 to 26.97)	30	−5.00 (−12.26 to 2.26)	23.44 (14.40 to 32.48)^b^	<.001	1.01
Implementation strategy							
12	40	7.05 (0.73 to 13.38)	40	0.07 (−4.13 to 4.27)	7.57 (−0.33 to 15.48)^b^	.06	0.45
18	31	12.68 (3.14 to 22.21)	38	−0.99 (−6.59 to 4.62)	12.16 (3.84 to 20.47)^b^	.004	0.54
24	27	23.30 (13.48 to 33.13)	30	0.97 (−6.21 to 8.16)	21.27 (12.37 to 30.17)^b^	<.001	0.87

Abbreviation: SAT-SF, Smart Management Strategy for Health Assessment Tool–Short Form.

^a^ Differences in score changes between groups and associated tests of effect were estimated by regression, adjusting for baseline covariates.

^b^ Effect sizes of at least 0.3 as standardized mean differences (Cohen *d*) were considered clinically relevant.

### Two-Year Overall Survival

**Figure 2.**
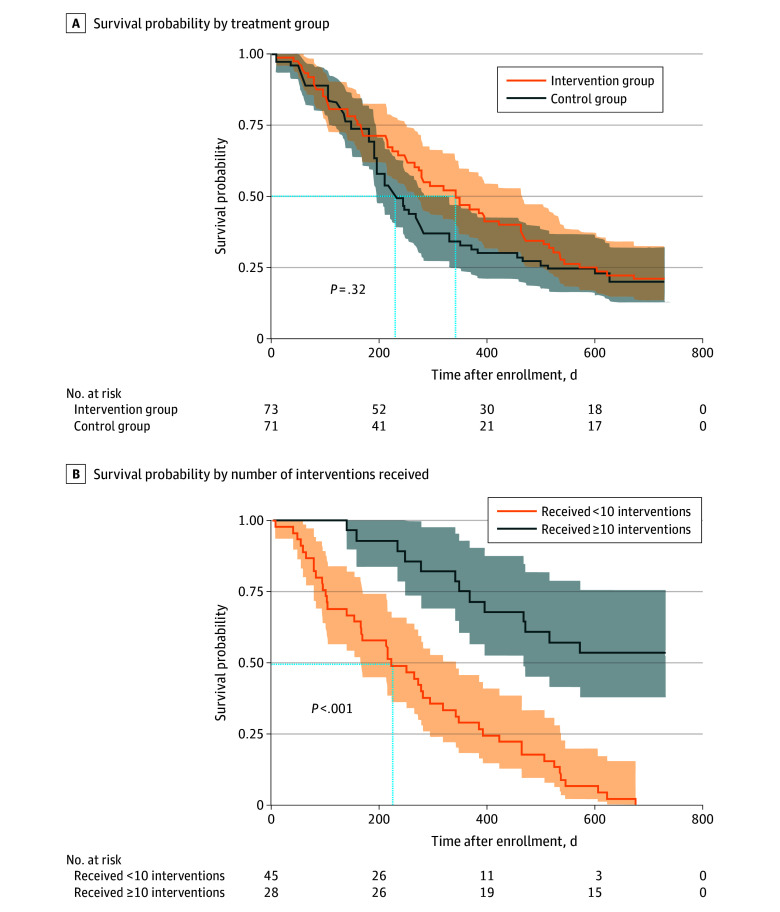
Kaplan-Meier Curve for Cancer Survival The early palliative care interventions included telephone coaching sessions and care team meetings and assessments. Shaded areas represent 95% CIs. The dashed blue lines represent the points with the most significant difference.

In the survival assessment, the number of survivors in the intervention group was high at both 200 and 400 days after enrollment, but there was no significant difference between the intervention and control groups (Figure 2A). However, when the number of EPC interventions (telephone coaching sessions and care team meetings and assessments) in the intervention group was classified for the survival comparison, the time point with the greatest survival difference was 221 days after the intervention (*P* < .001), with a significant and consistent difference in survival after enrollment. When divided by the number of EPC interventions, the 2-year survival probability increased significantly if the intervention was received 10 or more times (difference, 53.6%; *P* < .001) (Figure 2B).

## Discussion

This study confirmed that QOL improved more in participants receiving EPC at the 18-week interval; however, no significant improvements were observed at the 12- and 24-week intervals, which is similar to the findings of previous studies.^16,25,26^ . Furthermore, self-management strategy, including coping skills, also significantly improved with the EPC intervention. The change in self-management coping ability was evaluated in relation to the patient’s independent treatment decision-making, which is the goal of EPC.^1,27,28^ The survival rate increased significantly in the intervention group among those who received 10 or more EPC interventions (telephone coaching sessions and care team meetings and assessments), indicating that adherence with intensive intervention can increase the survival rate.

In previous studies, QOL substantially improved or deteriorated less in patients receiving EPC,^5^ but most studies confirmed the QOL improvement only in the short term of 12 weeks or demonstrated a small effect size.^16,18,26^ Considering that patients with advanced cancer may have a life expectancy of 24 weeks or more and that long-term improvement in QOL is important, QOL at 12, 18, and 24 weeks was assessed in this study along with existential and social burdens. This study showed that the score changes in various QOL measures, especially existential burden, were substantially better even after 24 weeks in the intervention group compared with the control group. These results suggest that the early introduction of PC after the diagnosis of advanced cancer leads to long-term improvement in QOL in patients with advanced cancer.

Additionally, we evaluated self-management strategies, which are still insufficiently studied, and found that EPC significantly increased self-management competency. One goal of the systematic EPC intervention in this study was to enhance patients’ self-management ability and coping skill. For this objective, nurse coaches encouraged patients to express freely how they would like to deal with problems and what they thought about their disease. Since a main purpose of EPC is self-directed treatment decision-making and life-sustaining decision-making,^18,29^ enhancing self-management competency can help patients with advanced cancer maintain a dignified QOL. If EPC interventions are properly provided, they can play a role in helping patients make independent decisions about their own lives.

In the case of survival rate, previous studies have reported that patients receiving EPC showed no significant difference in survival rate or that the effect size was too small even if the survival rate increased.^10,18^ In the present study, there was no significant difference in the survival rate of the group receiving EPC, but there was a significant difference in this group among those who received 10 or more interventions. This result shows the importance of not only introducing systematic interventions but also supporting patients to receive the offered intervention. It is possible that individuals in the intervention group with better general condition received more interventions; however, when EPC interventions are feasible, increased adherence to the intervention could potentially change the 2-year survival rate. Therefore, it might be beneficial to provide qualified interventions early on.

### Limitations

This study has several limitations. First, it was difficult to maintain an appropriate sample because patients were likely to become sicker over time. Nearly 40% of participants were lost to follow-up before 24 weeks after enrollment. A considerable number of patients dropped out early due to the perceived burden of the intervention. There were also substantial follow-up losses at 18 and 24 weeks, which may limit the interpretation of the results. In addition, the results were based on a relatively small sample because of recruitment difficulties. Second, these factors associated with pooling enough participants could have introduced selection bias. Although there were no significant differences in sociodemographic characteristics between the intervention and control groups, additional RCTs involving a larger number of participants may be needed in the future. Nonetheless, because the results are conceptually logical, it is unlikely they resulted from chance. Third, although this study confirmed that EPC interventions can improve QOL and enhance self-management competency, it did not confirm whether self-management competency affects decisions about life-sustaining treatment. Fourth, we tried to recruit patients with various cancer types and situations by conducting a multicenter RCT, but instead the study was limited by being conducted only in South Korea. We were unable to access all eligible patients; therefore, we relied on referrals from oncologists to recruit participants. Consequently, there was a possibility that the recruited patient cohort did not adequately represent the actual patient population. However, considering that most previous studies were conducted in the US and Europe, the present trial can be described as a major study confirming that EPC offers various benefits in Asia as well.

## Conclusions

Guidelines recommend EPC in the treatment of patients with advanced cancer. However, few studies have examined whether these EPC interventions affect long-term QOL and empower patients’ self-management capabilities. Although the present RCT showed that EPC did not improve QOL at 24 weeks, compared with the control group, participants in the intervention group reported significant improvement in self-management or coping skills. These results show the multifaceted benefits of EPC interventions. Moreover, this study confirmed that the 2-year survival rate was significantly higher among individuals in the intervention group who received 10 or more EPC interventions, indicating that increasing adherence is an important factor in these interventions. Based on these results, it may be necessary to develop guidelines for systematic EPC that would improve its quality in the long term. In addition, various methods of providing EPC also must be developed and applied to increase adherence with interventions, such as virtually or by telephone.

## Notes

Supplement 1.
Trial Protocol

Supplement 2. **eTable.** Mean Observed Change From Baseline and Difference in Change Relative to Baseline in MQOL (McGill Quality of Life Questionnaire) Between Groups

Supplement 3.
Data Sharing Statement
