# Supplementary material for: Early Integrated Palliative Care in Patients With Advanced Cancer: A Randomized Clinical Trial
Source: JAMA Netw Open. 2024 Aug 8;7(8):e2426304. doi: 10.1001/jamanetworkopen.2024.26304 (PMC11310828; doi:10.1001/jamanetworkopen.2024.26304)
Supplement: Supplement 1. — Trial Protocol [file jamanetwopen-e2426304-s001.pdf]

Protocol

Overview of the study **2**

Measurement and statistical analysis **5**

1) Measurement tools 5

2) Measurement procedure – Intervention Group 6

3) Measurement procedure – Control Group 8

4) Statistical analysis 9

Detailed study procedure **10**

1) Study registration and random assignment 10

2) Global assessment and PCT examination 12

3) Advance care planning (team meeting) 13

4) Follow-up 14

5) Telephone coaching 15

6) Crisis intervention (recommendation) 16

7) Interdisciplinary collaboration (recommendation) 17

8) Termination of service 18

9) Additional items 19

Overview of the study

Study Design and Participants

- **Type**: Nonblinded, randomized clinical trial.
- **Objective**: Evaluate standard supportive oncological care vs. early and systematic integrated palliative care in advanced cancer patients.
- **Exclusion**: Patients already receiving/had received palliative care.
- **Registration**: ClinicalTrials.gov (NCT03181854).
- **Duration**: September 2017 - October 2018.
- **Location**: 12 South Korean hospitals.
- **Recruitment**: Oncologists identified potential participants; both outpatients and inpatients were considered.

Inclusion Criteria:

1. Aged 20 or older.
2. Diagnosed with advanced solid tumor cancer (histologically/cytologically confirmed).
3. European Cooperative Oncology Group (ECOG) performance status: 0-2.
4. Estimated life expectancy of ≤12 months.
5. Consent to participate.

Exclusion Criteria:

1. Inability to speak/read Korean.
2. Poor health conditions, e.g., dyspnea, severe depression.
3. Not under current cancer treatment.
4. Received prior palliative care.

Approval: Institutional Review Board of Seoul National University College of Medicine and Hospital (H-1602-143-745).

Procedure

The clinical research coordinator (CRC) explained study details to eligible participants. Upon physician's recruitment, participants provided written informed consent.

Randomization

- **Method**: 1:1 ratio using a computerized random number generator (SAS 9·1·3; Proc plan).
- **Stratification**: Cancer type, age (< 65 or ≥ 65), and enrollment hospital.

Intervention

1. Initial meeting with palliative care team within three weeks post-randomization.
2. Self-study materials/videos provided on early palliative care/advance care planning (SMASH-based).
3. Telephone coaching: Weekly for the initial 12 weeks, then bi-weekly.
4. Symptom and psychological evaluation every three weeks for six months by the palliative care team.
5. Outpatient palliative care consultations according to a palliative care plan tailored to the patient’s needs.
6. Symptom assessment every three weeks via MDASI and PHQ-2.
7. Control group: Usual oncological care with optional usual palliative care.

Palliative Care Team

- Members: Palliative care specialist and health coach (nurses with >3 years clinical experience) - depending on the hospital, some teams also included social workers.
- Training: 23 hours offline lectures, 14 hours tele-class coaching practice, and 3 additional hours by International Coach Federation certified coaches.

Measures

- **Demographics**: Age, gender, education, employment, household income, residence.
- **Medical Information**: Tumor type/stage, ECOG status.
- **Primary Outcome**: Quality of life change over 24 weeks (via GHS/QOL scale of EORTC QLQ-C15-PAL).

- **Secondary Outcomes**: EORTC QLQ-C15-PAL functional/symptoms scales, MQOL functional scales, crisis-overcoming capacity (via SAT-SF).

Mortality Data Collection

Family caregivers provided data on deceased patients. The survival data obtained from Statistics Korea (KOSTAT).

Data Analysis

1) Survival analysis for two years post-enrollment using Kaplan-Meier curves.

2) Mean observed changes from baseline, adjusted differences in change scores at 12, 18, and 24 weeks were analyzed for survivors.

3) Effect sizes determined by standardized mean differences (Cohen's d), considering ≥ 0.3 as clinically relevant.

Measurement

1) Measurement tools

Measure	Baseline	Week 12	Week 18	Week 24
**1. Patient**				
General information	O			
Health-related quality of life indices<br>-EORTC QLQ-C15-PAL<br>-MQOL<br>-EQ-5D	O	O	O	O
Depression screening test (PHQ-9)	O	O	O	O
Disease understanding	O	O	O	O
Strategy survey (competence of overcoming crisis) (SAT-SF)	O	O	O	O
Preference of documenting advance directives for life-sustaining treatment	O	O	O	O
Quality assessment index (QCQ-EOL)	O	O		

Measure	Baseline	Week 12	Week 18	Week 24	3 months following patient death
**2. Family**					
General information	O				
Quality of life of patient's family (CQOL-C)	O	O	O	O	
Quality of life index (MQOL)					O
Depression screening test (PHQ-9)	O	O	O	O	O
Family's disease understanding	O	O	O	O	
Strategy survey (competence of overcoming crisis) (SAT-SF)	O	O	O	O	
Preference of documenting advance directives for life-sustaining treatment	O	O	O	O	
Cost-effectiveness analysis		O		O	
Complementary alternative medicine		O		O	
Quality assessment index (QCQ-EOL)					O

2) Measurement procedure – Intervention Group

(blue): PCT physician/ (red): CRC or PCT nurse/ (green): Both

INITIAL CARE	**1. Study registration and random assignment**	Once patients are referred by the treating department to register for the early palliative care research, they are briefly informed of the study and **asked to sign a written consent form**. Upon the completion of the Baseline Survey, **random assignment** is performed. Patients are provided with a copy of the early palliative care **leaflet and educational materials (on early palliative care and advance care planning)** and informed of the services to be provided later. **PCT team consultation is scheduled**.
	▼	
	**2. Registration and assessment prior to PCT examination**	Prior to the PCT team consultation, the CRC (or PCT nurse) meets patients assigned to the Intervention Group. **The Registration Form for Early Palliative Care Service, Global Assessment Form, and Summary Evaluation Form** are filled out.
	▼	
	**3. Examination by PCT physician and paperwork**	The PCT physician performs an **in-person examination** based on the completed Patient Registration Form and Summary Evaluation Form. After examination, a **Service Log entry** is made. **Subjects are registered on the website for the palliative care patient management program (PCT Web) and the Early Palliative Care Service Registration Form, Global Assessment Form, and Summary Evaluation Form are uploaded.**
	▼	
	**4. Establishment of advance care plan**	**At the weekly regular team meeting,** advance care plans are established for patients newly registered for early palliative care service. (One PCT physician and one CRC [or PCT nurse] must be in attendance.)
ROUTIN E CARE	**1. Follow-up**	The PCT physician performs regular in-person examinations (once every 3 weeks or once every 2 or 4 weeks if adjustment is necessary) by coordinating with the treating department's

		schedule. (In-person examination is conducted based on the Global Assessment Form and Summary Evaluation Form filled out prior to the examination.)<br>The CRC (or PCT nurse) **conducts surveys at weeks 12, 18, and 24**. After examination, the Global Assessment Form, Summary Evaluation Form, and Service Log **are uploaded to PCT Web**.
	▼	
	**2. Telephone coaching**	A nurse who completed coaching training conducts **telephone counseling** weekly from week 1 through week 12, and bi-weekly from week 13 through week 24, using the counseling workbook to manage symptoms and assess the need for crisis intervention. **A coaching log entry is made on the website for the palliative care patient management program.**
	▼	
	**3. Handover to the existing hospice team**	**If patients advance to an end stage and palliative care is the only treatment option, they are handed over to the conventional hospice team at the hospital** and **intervention is terminated**. Although the intervention for early palliative care is terminated, **follow-up surveys continue to be administered**.

	**Visit at week 24**	Upon completion of **the week-24 survey**, the study is completed.
	**In the case of patient death**	If a patient dies, the **Study Termination Form** is filled out.<br>**At 3 months after patient death, the family is contacted by phone** to administer the 3-Month Post-Death Survey.

(Recommendations)

CRISIS CARE	**1. Crisis intervention**	If a patient is admitted to ER, general ward, or intensive care unit, the CRC (or PCT nurse) visits the patient within 24 hours of admission, **examines the crisis situation, and makes a PCT consultation request** to the primary oncologist. The **PCT team meeting** should be held within 72 hours to establish a care plan (72 hours, i.e., 3 working days) is a recommendation). **Meeting**

		**minutes on care planning** are recorded and uploaded to the palliative care patient management program.
	▼	
	**2. Treatment consultation with other specialists**	If interdisciplinary care is necessary, **a request for treatment consultation is made to the appropriate specialists**.

3) Measurement procedure – Comparison Group

: PCT physician/ : CRC or PCT nurse

PROCEDURE	**1. Study registration and random assignment**	Once patients are referred by the treating department to register for the early palliative care research, they are briefly informed of the study and **asked to sign a written consent form**. Upon completion of the Baseline Survey, **random assignment** is performed. Patients are provided with a copy of the early palliative care **leaflet and patient educational materials (on cancer pain management)**.
	▼	
	**2. Follow-up**	At weeks 12, 18, and 24 **surveys will be administered**.<br>At follow-ups, **the materials (on advance care planning and early palliative care) developed for the study are provided**.

※ PCT team member roles may be modified in accordance with the situation at each institution.

4) Statistical analysis

- Main outcomes
  - The mean observed change from baseline and adjusted differences between change scores at 12, 18, and 24 weeks were analyzed based on the estimated regression model
  - Effect sizes were calculated as standardized mean differences (Cohen's d)
- Survival analysis
  - Two-year survival as the main survival outcome
  - Kaplan-Meier curves were used to compare the survival rates between the EPC and control groups two years after enrollment
  - To determine if the survival rate was higher in the group that received more interventions, participants were divided into groups with fewer than 10 interventions and more than 10 interventions
  - Patients who were alive at the time of the last follow-up (two years from the enrollment date) were censored on that date

Detailed study procedure

1) Study registration and random assignment

	Task	Actor	Details	Relevant Forms
**1**	Study introduction	Primary oncologist	■ Screen outpatients and inpatients on the inclusion and exclusion criteria.<br>■ Inform the patients who satisfy all inclusion criteria and do not meet any of the exclusion criteria, and their families, of the study in detail.<br>■ Instruct patients and their families who agree to participate in the study to sign a written consent form.<br>※ If the family did not accompany the patient to the hospital or does not wish to participate in the study, the patient can register for the study alone. However, if a patient refuses to participate, neither the patient nor the family can register.<br>※ For future analysis, patients' resident registration numbers will be collected and their consent on the utilization of the information will be obtained. The information will be used to make inquiries about patient deaths at Statistics Korea and to link to National Health Insurance Corporation databases to examine the rate of hospice care center utilization and conduct analyses on medical expenditure.<br>■ Study subjects are either instructed to contact CRC directly or guided to the palliative care outpatient clinic.	Study Description and Consent Form
**2**	Referral	Primary oncologist	■ At the time of referral, record the following information: *(recommendation)*<br>- Primary diagnosis (cancer type), purpose of the referral, current anticancer therapy, treatment plan if the current therapy fails	Palliative Care Patient Management Program<br>- Referral Form
**3**	Initial survey	CRC/PCT nurse	■ Administer the baseline survey.	Baseline Survey

**4**	Random assignment	CRC/PCT nurse	■ Connect to iCReaT to register patients and perform random assignments.<br>- Random assignment within an age group (<65 or ≥65)	iCReaT
**5**	Description of services to be provided	CRC/PCT nurse	■ Describe to patients the services they will receive.<br>- Intervention Group: Inform patients of the following: follow-up at weeks 12, 18, and 24; PCT examination approximately once every 3 weeks; and telephone coaching (weekly from week 1 through week 12 and bi-weekly from week 13 through week 24. Provide patients with copies of the workbook and the execution planner, and educational materials and CDs for early palliative care and advance care planning.<br>- Comparison Group: Inform patients of follow-up at weeks 12, 18, and 24, and provide them with educational materials and a CD on cancer pain management.<br>※ If patients in Comparison Group wish to receive palliative care, make an arrangement so that they may receive the existing conventional palliative care provided by the institution.	
**6**	(For Intervention Group)<br><br>Early palliative care registration and baseline assessment	CRC/PCT nurse	■ After patient registration, summarize the results and fill out the Basic Patient Registration Form.	Palliative Care Patient Management Program<br>- Basic Patient Registration Form
**7**	(For Intervention Group)<br>Scheduling appointments	CRC/PCT nurse	■ Schedule the next appointment for outpatient examination by a palliative care specialist on next hospital visit (or on a day of patient choice).	
**8**	Compensation Payment	CRC/PCT nurse	■ Ask patients to sign the Compensation Payment Form for their assigned group.<br>※After patients sign the form, send the form to the appropriate personnel at Seoul	

			University Hospital.	

※※※ **Comparison Between Intervention and Comparison Groups**

	Intervention Group	Comparison Group
**Materials to offer and the timing**	Baseline: Leaflet and CD on early palliative care<br>Leaflet and CD on advance care planning	Baseline: Leaflet on cancer pain management<br>Week 12: CD on cancer pain management<br>Week 18: Leaflet and CD on early palliative care<br>Week 24: Leaflet and CD on advance care planning
**Palliative care services to offer**	Services provided according to study protocol.<br>-PCT team consultation and examination<br>-Telephone coaching<br>-Crisis intervention<br>-Team meetings (to establish care plans)	Palliative care services provided by the institution.

2) Global assessment and PCT examination

Task		Actor	Details	Relevant Form
**1**	Global assessment	CRC/PCT nurse	■ Meet patient prior to outpatient examination, and perform global assessment for palliative care need (Global Assessment Form to be filled out by patient). Summarize the results and fill out Summary Palliative Care Evaluation Form.<br>■ Provide PCT physician with copies of Basic Patient Registration Form, Summary Palliative Care Evaluation Form, and Service Log.	Palliative Care Patient Management Program<br>- Global Assessment Form<br>- Summary Evaluation Form
**2**	In-person examination	PCT physician	■ Review Basic Patient Registration and Summary Palliative Care Evaluation Forms (if necessary, review Global Assessment Form as well).<br>■ Ask questions and perform a physical examination to determine whether the patient needs palliative care, disease understanding, and advance care planning. Make a global assessment and identify patient need for care.	Palliative Care Patient Management Program<br>-Basic Patient Registration Form<br>-Summary

			■ Explain future care plan to the family.<br>■ Issue an order for symptom management, if the patient manifests a symptom requiring an immediate intervention. (If necessary, make a request for assessment and intervention to other medical departments such as psychiatry or social work.)<br>■ Record the findings in the electronic medical records (EMR).	Evaluation Form
**3**	Summarization	PCT physician, CRC/PCT nurse	■ Check the counseling and care items provided to the patient in the Service Log.<br>■ If there is a crisis notification on the EMR Alert/Remind window (e.g., ER admission), inform the PCT nurse or CRC, and make a record to refer to the PCT team. *(recommendation)*	Palliative Care Patient Management Program<br>-Service Log

3) Advance care planning (team meeting)

Task		Actor	Details	Relevant Forms
**1**	Team meeting	PCT team (PCT physician, PCT nurse, social worker, etc.)	■ During weekly regular team meetings, establish advance care plans for patients registered in the past week.<br>■ Discuss patients for whom the care plan should be modified or an interdisciplinary crisis intervention is needed, among those for whom advance care plans were established and care has been provided.<br>- Review global assessment findings from multiple angles<br>• Basic Patient Registration Form<br>• Summary Evaluation Form<br>• Initial and recently revised Case Report Forms<br>• Outpatient exam records	Palliative Care Patient Management Program<br>- Basic Patient Registration Form<br>- Summary Evaluation Form<br>- Case Report Form<br>- Outpatient Exam Records
**2**	Summarization	CRC/PCT nurse	■ Document advance care plans by summarizing the discussions, and share with	Palliative Care Patient Management

			team members.	Program<br>-Meeting Minutes on Care Planning

4) Follow-up

Task		Actor	Details	Relevant Forms
**1**	Preparation for examination	CRC/PCT nurse	■ Fill out Summary Evaluation Form based on the Global Assessment Form completed by patients on the same day and the telephone counseling sessions over the previous period.<br>■ Provide the PCT physician with copies of the Basic Patient Registration Form and Summary Evaluation Form.	Palliative Care Patient Management Program<br>-Global Assessment Form<br>- Summary Evaluation Form
**2**	In-person examination	PCT physician	■ Review Summary Evaluation Form. (If necessary, review Global Assessment Form, as well.)<br>■ Determine whether symptoms observed in the last outpatient examination are under control.<br>■ Globally assess the patient by asking additional questions and performing a physical examination, and identify care needs.<br>■ Issue an order for symptom management.<br>■ If necessary, make a consultation request to other medical departments or the social work department for evaluation and intervention.<br>■ Record examination findings in the EMR.	
**3**	Summarization	PCT physician/PCT nurse	■ Record counseling and care items in Service Log.	Palliative Care Patient Management Program<br>- Service Log

**4**	Follow-up survey	CRC	• Administer follow-up surveys at weeks 12, 18, and 24.<br>• If a patient dies during the study period, contact the family by phone 3 months after the patient's death to administer a follow-up survey.	- Week 12, 18, and 24 Surveys<br>-3-Month Post-Death Survey

5) Telephone coaching

Task		Actor	Details	Relevant Forms
**1**	Telephone coaching	Health coach (should have completed coaching training)	■ Review Summary Evaluation Form and the outpatient examination records.<br>■ Prior to coaching, confirm the following items:*(recommendation)*<br>• Whether moderate/severe symptoms observed in the previous physical exam have improved<br>• Whether new symptoms/problems have occurred<br>■ Address a crisis situation requiring intervention, in the following manner: *(recommendation)*<br>• If the patient urgently needs to go to the hospital, make an appointment at the PCT outpatient clinic for the same or next day and inform the patient.<br>• If the patient needs to go to the ER, arrange an ER visit and notify the PCT physician.<br>• If it is difficult to determine, ask the PCT nurse to contact the primary oncologist and follow the physician's instructions.<br>■ Go over with patients the step-by-step strategy to cope with crises during the journey of cancer in sequence.<br>■ Assign coaching tasks for patients to perform until the next session and encourage them.<br>■ Record the counseling session and care items in the Telephone Counseling Log.<br>※ The indications requiring hospital admission are determined based on the anticancer education materials provided by the medical oncology and	Palliative Care Patient Management Program<br>- Telephone Coaching Log

			hematology departments of the institution.<br>※ The PCT physician may create a primary doctor or fellow hotline, depending on the hospital situation.	

6) Crisis intervention (recommendation)

- If one of the following crisis situations occurs in patients who were registered for the program and has undergone the baseline assessment, establish a palliative care plan and provide care:

(1) ER admission

(2) Hospital admission due to an unexpected cause other than for scheduled short-term anticancer therapy

(3) intensive care unit admission

※ (1) is limited to the cases in which the reason for ER admission is cancer-related. Example: A patient with gastric cancer admitted to the ER for minor trauma is not considered under (1).

※ With regard to (2), a patient who is admitted to the hospital for short-term anticancer therapy but needs to stay at the hospital beyond the scheduled length of stay due to another issue is also regarded as a case requiring crisis intervention. Example: if a patient is admitted to the hospital for anticancer therapy, but hospital stay is prolonged because of a delay in anticancer treatment due to a need for infection control following the occurrence of pneumonia before or during the stay, or hospital stay is prolonged to control the infection even after the completion of anticancer treatment; if a patient is admitted to the hospital for anticancer therapy but required to fast due to bowel obstruction, which prevents anticancer treatment and prolongs hospital stay.

Task		Actor	Details	Relevant Forms
**1**	Crisis recognition	EMR system	■ Each research hospital establishes a system to recognize an occurrence of crisis.<br>- Exemplar approaches to facilitate the recognition of a need for crisis intervention:<br>• During the first in-person examination, instruct the family to contact the PCT nurse if a crisis such as ER admission occurs.<br>• If a crisis notification appears in the EMR Alert/Remind window (e.g., ER admission), contact the PCT nurse or CRC and make a note to consult with	

			the PCT team.<br>• Prior to a regular team meeting, once or twice a week, perform a screening to check on hospital admission of any patients registered for the early palliative care program.	
**2**	Summarization of basic information	PCT nurse	■ Visit the patient, globally assess the type and cause of the crisis and need for palliative care, and identify patient need for care, within 24 hours (of working days).<br>■ Examine the patient's and family's perceptions of and attitudes toward the crisis; inquire whether advance care planning was discussed and documented; and confirm whether they are willing to prepare for an advance care plan depending on the situation.<br>■ Request the primary oncologist to issue a consultation request to the PCT team.	Summary Crisis Assessment Form
**3**	Crisis intervention	PCT team meeting	■ It is recommended to hold a PCT team meeting within 72 hours (of working days).<br>■ Review the Summary Crisis Assessment Form; globally assess patient need for palliative care, disease understanding, and advance care planning; and establish a care plan.<br>■ Explain the care plan to the patient and the family, and provide the care.	Summary Crisis Assessment Form
**4**	Summarization	PCT physician/PCT nurse	■ Record the counseling items and care services using the Service Log checklist.	Service Log

7) Interdisciplinary collaboration (recommendation)

- If during baseline assessment or follow-up, the PCT physician determines that there is a need for interdisciplinary care, he or she can request the cooperation of other departments.

- Cases requiring collaboration with other disciplines are handled as follows:
  - Uncontrolled pain → anesthesiologist
  - Mental symptoms such as depression, delirium, anxiety, and sleep disturbances → psychiatrist
  - Need for dietary change → nutritionist
  - Need for spiritual care → priest
  - Need for clinical ethics consultation → Clinical Research Ethics Center

8) Termination of service

- Termination of service is defined as the termination of early palliative care service. Survey data collection continues until study completion.
- If early palliative care service is terminated in a patient, the final team meeting is held the same week and a record is made in the revised care plan (termination of service).
- Service is terminated in one of the following cases:
  - Patient death
  - Drop out (due to a cause other than death) or withdrawal from the study

    Record the reason for withdrawal. (Note status change in Case Report Form and iCReaT.)

  - If anticancer therapy cannot be performed any longer and palliative care is the only option

    Oncologist consults with the hospice center. Subsequent hospice palliative care will be performed as conventionally provided by each research hospital.

9) Additional items

- PCT tasks for early palliative care may be modified in accordance with the situation of each research hospital. However, the research team must include at least one PCT physician (part-time PCT physician allowed but training must have been completed according to the protocol) and at least one PCT nurse (PCT nurse must have completed training according to the protocol). If he or she has completed training according to the protocol, a member of PCU may take additional responsibilities. However, the research team must include at least one full-time PCT nurse.
- If a social worker regularly performs the care tasks of the existing palliative care team at the research hospital, some nursing care tasks in the early palliative care program can be performed by the social worker.
