# Supplementary material for: Early Integrated Palliative Care in Patients With Advanced Cancer: A Randomized Clinical Trial
Source: JAMA Netw Open. 2024 Aug 8;7(8):e2426304. doi: 10.1001/jamanetworkopen.2024.26304 (PMC11310828; doi:10.1001/jamanetworkopen.2024.26304)
Supplement: Supplement 2. — eTable. Mean Observed Change From Baseline and Difference in Change Relative to Baseline in MQOL (McGill Quality of Life Questionnaire) Between Groups [file jamanetwopen-e2426304-s002.pdf]

Supplemental Online Content

Kang EK, Kang JH, Koh SJ, et al. Early integrated palliative care in patients with advanced cancer: a randomized clinical trial. *JAMA Netw Open*. 2024;7(8):e2426304. doi:10.1001/jamanetworkopen.2024.26304

eTable. Mean Observed Change From Baseline and Difference in Change Relative to Baseline in MQOL (McGill Quality of Life Questionnaire) Between Groups

This supplemental material has been provided by the authors to give readers additional information about their work.

© 2024 Kang EK et al. *JAMA Network Open*

eTable. Mean observed change from baseline and difference in change relative to baseline in MQOL (McGill Quality of Life Questionnaire) between groups

Measure	Intervention		Control		Available cases analysis†		
Time from baseline (months)	N	Mean observed change from baseline (95% CI)	N	Mean observed change from baseline (95% CI)	Adjusted difference between change scores (95% CI)	p value	Effect size
Existential Well-Being							
12 weeks	40	0.07 (-0.65 to 0.79)	40	0.14 (-0.43 to 0.72)	-0.28 (-1.03 to 0.47)	0.46	-0.14
18 weeks	31	0.62 (-0.02 to 1.27)	38	-0 .15 (-0.72 to 0.42)	0.38 (-0.41 to 1.18)	0.35	0.21
24 weeks	27	0.62 (-0.31 to 1.55)	30	-0.42 (-1.01 to 0.17)	0.82* (0.03 to 1.67)	0.05	0.4
Social Support							
12 weeks	40	-0.23 (-0.86 to 0.41)	40	0.03 (-0.69 to 0.74)	-0.28 (-1.03 to 0.47)	0.47	-0.13
18 weeks	31	0 (-0.65 to 0.65)	38	-0.47 (-1.02 to 0.07)	0.31 (-0.49 to 1.11)	0.45	0.18
24 weeks	27	0.19 (-0.57 to 0.94)	30	-0.68 (-1.36 to -0.01)	0.65* (-0.21 to 1.51)	0.14	0.34

* Effect sizes of at least 0.3 as standardized mean differences (Cohen's d) were considered clinically relevant.
† Differences in change scores between groups and associated tests of effect were estimated by regression, adjusting for baseline covariates.

© 2024 Kang EK et al. *JAMA Network Open*
