# Supplementary material for: Early Integrated Palliative Care in Patients With Advanced Cancer: A Randomized Clinical Trial
Source: JAMA Netw Open. 2024 Aug 8;7(8):e2426304. doi: 10.1001/jamanetworkopen.2024.26304 (PMC11310828; doi:10.1001/jamanetworkopen.2024.26304)
Supplement: Supplement 3. — Data Sharing Statement [file jamanetwopen-e2426304-s003.pdf]

Data Sharing Statement

Kang. Early Integrated Palliative Care in Patients With Advanced Cancer. *JAMA Netw Open*.
Published August 08, 2024. doi:10.1001/jamanetworkopen.2024.26304

Data
Data available: Yes
Data types: Deidentified participant data
How to access data: lawyun08@gmail.com
When available: With publication

Supporting Documents
Document types: None

Additional Information
Who can access the data: researchers whose proposed use of the data has been approved
Types of analyses: research purpose only
Mechanisms of data availability: after approval of a proposal
